# Research on Laser Direct Transmission Welding of Transparent Polystyrene and Polycarbonate Based on Laser Surface Modification

**DOI:** 10.3390/polym17030409

**Published:** 2025-02-04

**Authors:** Kehui Zhai, Fuhao Yang, Qiyan Gu, Yu Lin, Minqiu Liu, Deqin Ouyang, Yewang Chen, Ying Zhang, Qitao Lue, Shuangchen Ruan

**Affiliations:** 1 Key Laboratory of Advanced Optical Precision Manufacturing Technology of Guangdong Higher Education Institutes, Sino-German College of Intelligent Manufacturing, Shenzhen Technology University, Shenzhen 518118, China; zkh98210@163.com (K.Z.); yyangfuhao@163.com (F.Y.); 2210412034@stumail.sztu.edu.cn (Q.G.); 2450266002@stumail.sztu.edu.cn (Y.L.); ouyangdeqin@sztu.edu.cn (D.O.); chenyewang@sztu.edu.cn (Y.C.); zhangying2@sztu.edu.cn (Y.Z.); lueqitao@sztu.edu.cn (Q.L.); scruan@sztu.edu.com (S.R.); 2 College of Applied Technology, Shenzhen University, Shenzhen 518060, China; 3 Shenzhen Key Laboratory of Laser Engineering, Guangdong Provincial Key Laboratory of Micro/Nano Optomechatronics Engineering, College of Physics and Optoelectronic Engineering, Shenzhen University, Shenzhen 518060, China

**Keywords:** laser transmission welding, laser surface modification, welding performance, absorbent free, miscibility

## Abstract

The conventional near-infrared laser transmission welding (LTW) process for joining dissimilar transparent polymers is limited by the need to incorporate optical absorbents, which compromises joint performance and raises biocompatibility concerns. To address these issues, this study proposed a surface modification technique using femtosecond laser ablation prior to the welding process. Experiments involved 520 nm femtosecond laser ablation of transparent polymers, followed by LTW of dissimilar transparent polymers using an 808 nm laser, with subsequent characterization and mechanical property evaluations. A maximum joint strength of 13.65 MPa was achieved. A comprehensive investigation was conducted into the physical and chemical mechanisms through which laser ablation improved the welding performance of dissimilar transparent polymers. The results demonstrated that laser ablation generated microstructures that serve as substitutes for optical absorbents while also facilitating the formation of numerous oxygen-containing functional groups. These enhancements improve miscibility and bonding performance between dissimilar polymers, enabling absorbent-free welding between ablated polycarbonate (PC) and polystyrene (PS). This work confirms both the feasibility and potential application of this process for direct LTW of dissimilar transparent polymers.

## 1. Introduction

Transparent polymers are extensively utilized in the chemical and manufacturing industries due to their high light transmittance, low density, excellent insulation properties, favorable mechanical performance, and shatter resistance. These materials can be conveniently processed into various forms, such as transparent sheets, tubes, rods, and other shapes suitable for a wide range of applications. In practical applications, the diverse properties of polymers—such as varying chemical resistance and mechanical strength—often necessitate the combination of different types of plastics. Currently, the joining of dissimilar polymers and their composites primarily relies on three methods: mechanical fastening [1], adhesive bonding [2], and welding. However, mechanical fastening can cause stress concentration issues, while adhesive bonding often suffers from low efficiency and susceptibility to aging. In contrast, welding processes are considered superior techniques for joining polymer composites. Among these techniques, LTW stands out for its efficiency, precision, cleanliness, and vibration-free characteristics. As a result, LTW is gradually emerging as the preferred technique for joining polymer composite materials across various applications.

The vibrational harmonics, or composite absorption spectra, of organic polymer chains occur at wavelengths exceeding 1500 nm [3]. Consequently, the inherently weak absorption of near-infrared light by pure polymers poses challenges in directly welding transparent polymers, regardless of whether they are similar or dissimilar. This issue represents a significant obstacle in the field of LTW. Currently, commonly used LTW light sources include lasers with wavelengths such as 793 nm, 808 nm, and 1064 nm [4]. However, these wavelengths present difficulties when applied directly to welding transparent polymers. To improve laser light absorption in transparent weldments—whether composed of similar or dissimilar materials—traditional methods often involve applying an absorbent coating to the surface before welding or incorporating absorbents during the injection molding process. Common absorbents include carbon black [5], Clearweld [6], metals, and their oxides [7,8,9]. In recent years, efforts have focused on exploring novel materials, such as cold-sputtered metals [10] and highly polar polymer grafting [11], as innovative light absorbers. Research has primarily aimed to evaluate the impact of these new materials on laser light absorption and their subsequent influence on the joint performance of welded samples.

The welding method incorporating absorbents has successfully achieved high-strength joints between transparent polymers and is currently the predominant technique for polymer LTW. However, this process often causes discoloration in the weld area, significantly compromising the aesthetic quality of transparent welded components. Additionally, some absorbents are non-biocompatible and pose potential health risks [12], which limits the applicability of the technique in fields such as biology, food safety, and medicine. Additionally, the uniformity of the absorbent coating on the weld surface is critical for ensuring connection quality; however, achieving consistent coatings typically requires advanced manual skills or specialized equipment [4], which increases costs and further restricts the application and promotion of LTW. To address these challenges, researchers have proposed using mid-infrared lasers with higher polymer absorption rates as an alternative light source for LTW [13,14]. Extensive experimental studies have been conducted on this approach [15]. In 2012, Mingareev et al. [16] performed pioneering LTW experiments on polymers such as PMMA, POM, PP, and PETG without absorbents, using a laser with a 2 μm wavelength. They obtained satisfactory welding results. Subsequent studies demonstrated that other materials, including ABS, PC, and PS [17], can also be directly welded using lasers at a 2 μm wavelength. The heating mechanism for 2 μm laser welding is characterized by typical top-to-bottom volume absorption; however, the high absorption rate may cause thermal damage to the polymer weldment surface, resulting in degradation of the plastic layer and reduced aesthetic quality [16]. To mitigate surface thermal damage during the welding process, transparent glass with high transmission and good thermal conductivity at a 2 μm wavelength—commonly far-infrared fused quartz—is often used as a heat sink positioned above the weldment surface. However, the inclusion of heat dissipation glass complicates the process and is primarily suitable for plate-like weldments. Furthermore, volume absorption may induce significant internal stress within the weldment, which can adversely affect connection strength [15,18]. These limitations have consequently restricted the broader application of 2 μm lasers in polymer welding.

Achieving good miscibility between dissimilar polymers is crucial for successful fusion and bonding. Most dissimilar polymer pairs exhibit poor miscibility [19], which makes direct welding highly challenging. To address this issue, researchers have explored various surface modification techniques, such as chemical grafting, plasma surface treatment, and cold metal sputtering [10]. These approaches have enabled effective connections in subsequent welding processes of dissimilar polymers. However, the connection strength achieved using these enhanced techniques generally remains low (<10 MPa), limiting their applicability for high-strength welding. Moreover, these surface treatments are often inefficient, leading to increased processing costs, while precise control of the treated surface area poses significant challenges [14]. Consequently, implementing these methods in practical large-scale industrial applications is difficult.

Laser surface treatment processes are renowned for their cleanliness, efficiency, and controllability. These methods have been widely employed for polymer surface modification to achieve functional properties, such as superhydrophobicity [20], biocompatibility [21], and adhesion [22,23]. Additionally, laser techniques are used to enhance the welding performance of dissimilar polymers. For example, in 2023, Liu et al. [24] pioneered the use of ultraviolet femtosecond laser ablation to modify the surface of polycarbonate (PC). Using this modified surface, they directly welded PC/GFRP with a heat-dissipation glass and a 2 μm laser, achieving an impressive bond strength of 13.7 MPa. This result highlights the significant impact of laser surface treatments on improving the welding performance of dissimilar polymers.

This study proposes the application of femtosecond laser surface treatment to enhance the direct welding performance of dissimilar transparent polymers. On one hand, laser surface treatment induces the formation of nano- or submicron-scale structures on the polymer surface, thereby improving laser light absorption [25]. The modified surface area effectively substitutes conventional light absorbents, enabling direct welding of dissimilar transparent polymers using near-infrared lasers (808 nm). On the other hand, this process generates abundant oxygen-containing functional groups on the polymer surface, which enhances miscibility and welding performance, ultimately increasing the joint connection strength. The objective of this work is to demonstrate the feasibility of using femtosecond laser surface treatment in LTW as a replacement for traditional light absorbents while improving connection performance between dissimilar transparent polymers. Furthermore, the study aims to elucidate the underlying physical and chemical mechanisms. Experimental results reveal that the laser ablation process not only effectively replaces light absorbents in LTW but also significantly enhances the connection performance between dissimilar transparent polymers.

## 2. Materials and Methods

### 2.1. Materials

PC is well known for its outstanding impact resistance, transparency, and thermal stability. It is widely employed in the manufacturing of transparent components, including applications such as electronic device housings, building materials, and eyeglass lenses. In contrast, polystyrene (PS) offers notable advantages such as excellent transparency, toughness, corrosion resistance, and ease of processing. These properties make it a suitable material for food packaging, electronic housings, consumer goods, and medical devices. This study investigates unweldable pure PC and PS as the primary materials. Both materials are specified with dimensions of 50 × 25 × 2 mm^3^, and some of their physical properties are listed in Table 1. The transmittance was measured using a UV–visible–NIR spectrophotometer (model: Lambda50+).

### 2.2. Femtosecond Laser Surface Modification

**Figure 1:**
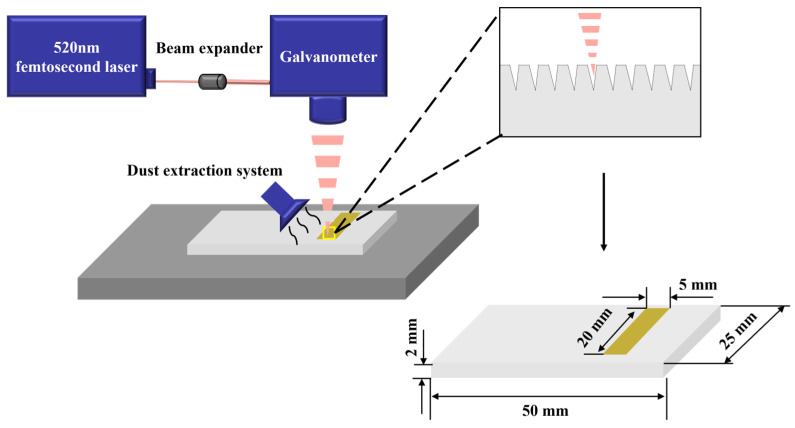
Schematic diagram of femtosecond laser ablation of polymer.

The femtosecond laser processing system and the corresponding ablation process flowchart are shown in Figure 1. The ablation light source is a femtosecond laser with a wavelength of 520 nm, a maximum average output power of 8 W, a repetition rate of 250 kHz, and a pulse width of 300 fs. The laser is controlled using a high-precision galvanometer scanning system, and the beam is focused through a field lens to produce a spot diameter of approximately 20 μm. The sample is placed on the worktable, with the laser focus adjusted to maintain a defocus distance of 0 mm from the upper surface of the sample. The ablation area is defined as 5 × 20 mm^2^, and a fill-scanning method is employed. During ablation, the laser scanning speed is set to 2500 mm/s, with an inter-line spacing of 30 μm and six repetitions [24,26]. To investigate the effects of different powers on ablation, the laser power (P_Ablation_) is varied from 1 to 8 W in increments of 1 W. Before ablation begins, contaminants such as mold release agents or dust are removed from the sample surface using ethanol with a purity of 99.99%. A vacuum cleaner is positioned on one side of the sample to capture dust particles and gases generated during the ablative process, thereby minimizing reflection, scattering, and absorption caused by interference from the ablation plume.

### 2.3. Near-Infrared Laser Welding Experiment

**Figure 2:**
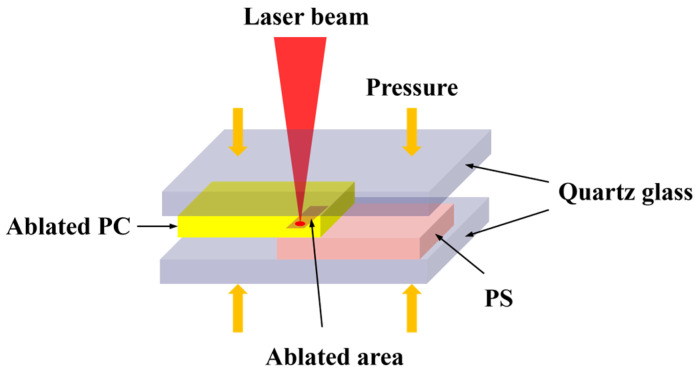
Laser welding experiment schematic diagram.

The ablated PC and PS materials were stacked and placed between two quartz glass plates, which exhibit high transmittance for 808 nm laser radiation (as shown in Figure 2). A pair of cylinders, each with a 25 mm diameter, equipped with guide rods and pneumatic actuators, was used to enable the vertical movement of the lower glass plate while maintaining a constant air pressure of 0.4 MPa. The welding light source was an 808 nm semiconductor laser with a maximum output power of 50 W and a minimum beam spot size of 800 μm. During the welding process, the defocus distance between the beam spot and the welding surface was maintained at 0 mm, and the welding speed was set to 6 mm/s using a single linear contour welding method. To assess the bonding strength under different laser powers, the welding power (P_Welding_) was varied from 5.5 W to 7.5 W in 0.5 W increments.

### 2.4. Characterisation and Mechanical Property Testing

To assess the light absorption and surface roughness of the samples before and after ablation, UV–visible–NIR spectrophotometry was performed on ablated PC and PS samples to measure transmittance in the wavelength range of 300 to 2200 nm. The surface roughness (Sa) of the ablated samples was evaluated using a laser scanning confocal microscope (LSCM). Scanning electron microscopy (SEM) was employed to image and analyze the microstructure of the ablated regions. X-ray photoelectron spectroscopy (XPS) and Fourier-transform infrared spectroscopy (FTIR) were used to examine the chemical composition of the sample surfaces both before and after ablation, allowing for an assessment of chemical changes on these surfaces. A contact angle meter was utilized to measure the water contact angle (WCA) with a droplet volume of 2 μL before and after ablation to investigate changes in surface wettability. Following welding, an optical microscope (OM) was used to observe the weld seam microstructure and measure its width (B). To quantify the weld connection strength, shear strength tests were conducted on the welded samples using a universal tensile testing machine at a constant moving speed of 2 mm/min. The maximum destructive shear force sustained by each sample was recorded, and shear strength values were subsequently calculated using Equation (1).

(1) σ=F/(L×B)

In the equation, σ denotes the shear strength (MPa), F is the shear force (N), L is the weld seam length (20 mm, a fixed value), and B represents the weld seam width (mm).

## 3. Results and Discussion

### 3.1. Laser Surface Modification Experiment Results and Analysis

The ultrahigh intensity makes femtosecond laser-material interactions a strongly nonlinear process. The intensities of femtosecond lasers can easily exceed 10^12^ W/cm^−2^, facilitating the occurrence of nonlinear ionization mechanisms during femtosecond laser fabrication processes, which means transparent polymers can exhibit nonlinear absorption to ultrafast lasers. Notably, the nonlinear ionizations are almost independent of the initial defects of the target materials [27]. Consequently, a 520 nm femtosecond laser can effectively be employed to process PC surfaces, despite PC samples exhibiting relatively high transmittance at this wavelength.

**Figure 3:**
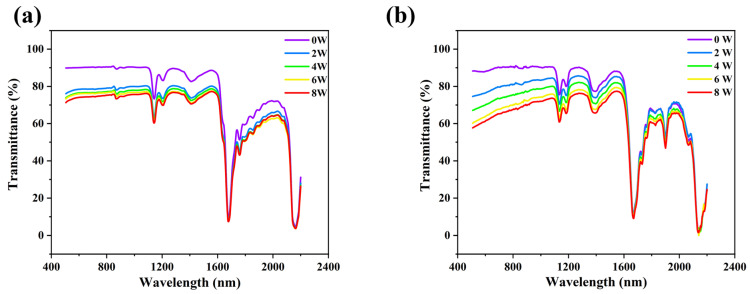
Transmittance changes in (**a**) PS and (**b**) PC after treatment with different femtosecond laser powers.

The relationship between the transmittance of PC and PS as a function of ablation power is presented in Figure 3a,b, respectively. Overall, as the ablation power increases from 0 W to 8 W, the transmittance of both PC and PS decreases progressively across various wavelength ranges. Specifically, at an ablation power of 8 W, the transmittance at a wavelength of 808 nm is measured at 67% for PC and 75% for PS. The absorption rate of the materials with respect to light is generally described by Equation (2):

(2) 1=A+T+R

Under the assumption that minor variations in reflectance (R) are negligible, a lower transmittance (T) corresponds to a higher absorption rate (A). It can therefore be inferred that increasing ablation power enhances the absorption rates of PC and PS, thereby improving laser absorption in LTW. Preliminary laser welding experiments revealed that ablated PC could successfully bond with pristine PS; however, the bonding between ablated PS and PC was unsuccessful. Accordingly, this study focuses on PC for further investigation in laser modification experiments.

**Figure 4:**
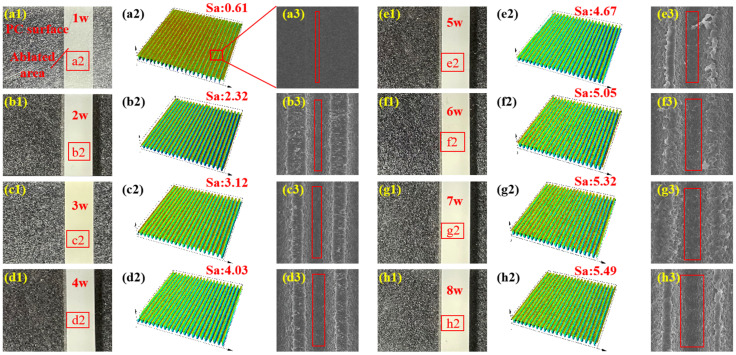
Surface roughness and micro-morphology of PC under different laser ablation powers: (**a1**–**h1**) charge-coupled device (CCD) camera; (**a2**–**h2**) laser scanning confocal microscope (LSCM); (**a3**–**h3**) scanning electron microscopy (SEM).

**Figure 5:**
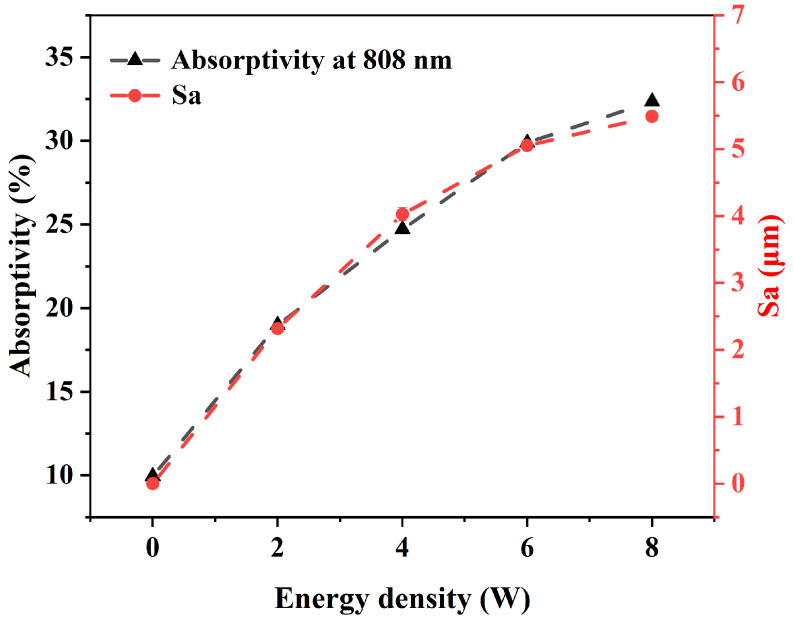
Plot of the relationship between the surface roughness and transmittance of PC with the ablation laser power.

In ultrafast laser processing, the peak power of the laser is extremely high, enabling the instantaneous vaporization of material within the affected region. Because the pulse duration is shorter than the timescale of electron–phonon interactions, the energy deposited in the electron gas cannot transfer effectively to the surrounding atoms or lattice structures. As a result, this energy is carried away with the vaporized material as soon as the laser irradiation ends at the conclusion of each pulse. The primary morphological features of ultrafast laser processing include a minimal heat-affected zone and well-defined processing boundaries [20,28]. In this study, sub-micrometer-scale grooves were fabricated on PC surfaces by repeated scanning with a 520 nm femtosecond laser (as shown in Figure 4). As the ablation power increased, both the width and depth of these grooves expanded correspondingly (refer to red squares in Figure 4a3–h3). Measurements of surface roughness and transmittance at 808 nm for the ablated samples revealed a positive correlation between these two parameters and laser power. The trends in surface roughness and transmittance curves were nearly identical, indicating a strong positive relationship between transmittance and surface roughness (as shown in Figure 5). Since the micro-morphology of PC under different laser ablation powers had the same spacing (Figure 4), the surface roughness was mainly determined by their depth. As the ablation power increased, there was a corresponding rise in roughness, indicating an increase in the depth of the micro-morphology. This enhancement created favorable conditions for the material’s surface to absorb more light effectively. Therefore, by adjusting the ablation laser power, it is possible to effectively control the surface roughness of the material while achieving the desired transmittance levels in processed samples.

**Figure 6:**
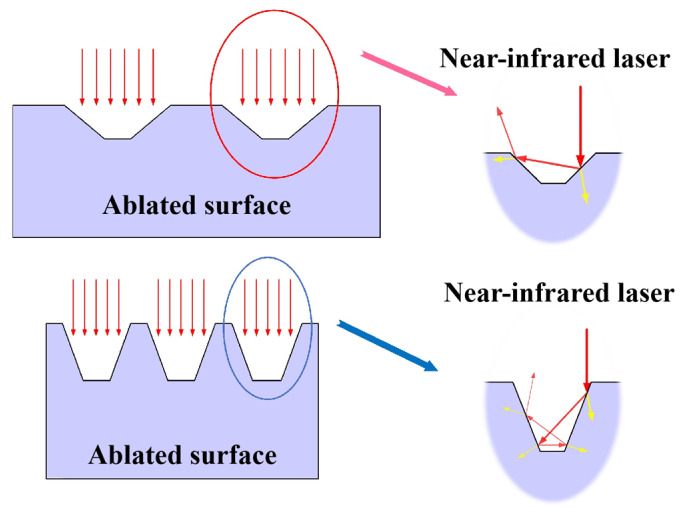
Schematic diagram of the effect of surface micro-morphology on material light absorption.

According to Equation (2), the reduction in transmittance after ablation can be attributed to increased light absorption. The absorption capability of the material is closely related to the surface micro-morphology, which can be characterized in three main aspects: (a) when the light beam interacts with the inner walls of surface micro-grooves, the angle of incidence decreases, leading to enhanced absorption [29]; (b) the groove structure increases the specific surface area of the material, allowing more laser energy to be absorbed per unit surface area; and (c) within the inverted triangular configuration of the grooves, the light beam undergoes multiple reflections and absorptions, facilitating repeated interactions with the material that significantly enhancing overall light absorption intensity. This phenomenon is illustrated in the schematic diagram in Figure 6. In this study, as the ablation power increased, wider and deeper micro-grooves were formed, creating favorable conditions for enhanced light absorption on the surface of the material. Therefore, changes in surface micro-morphology are a key factor in effectively regulating absorption rates of the material through laser surface modification processes. The resultant increase in absorption rate supports the replacement of absorbents in transparent polymers for LTW applications.

**Figure 7:**
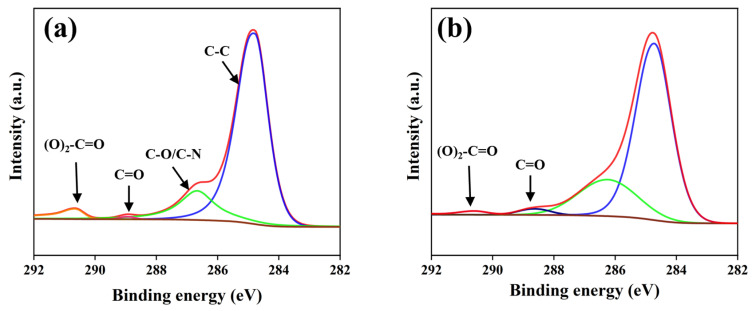
XPS spectra of PC: (**a**) before modification; (**b**) after modification P_Ablation_ = 8 W.

**Figure 8:**
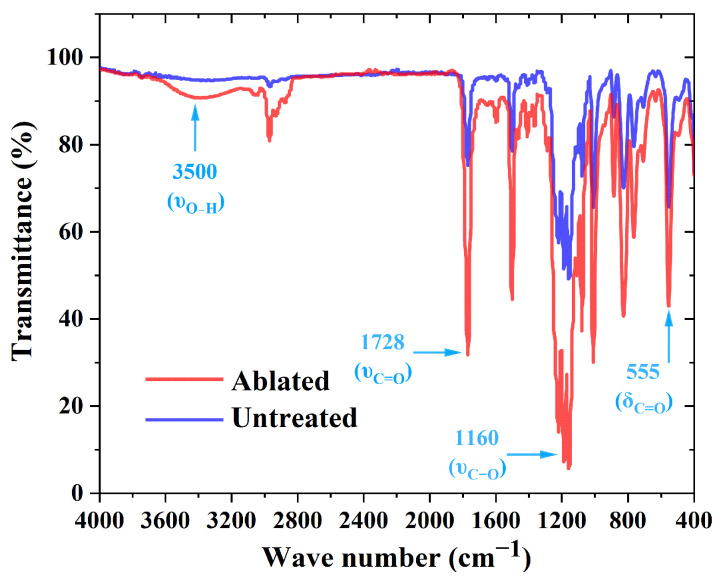
FTIR spectra of PC before and after ablation, P_Ablation_ = 8 W.

**Figure 9:**
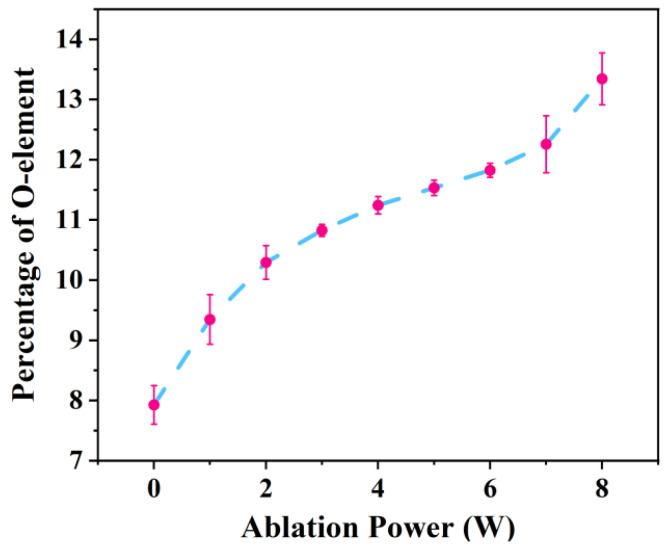
Oxygen content on the surface of PC under different ablation powers.

**Figure 10:**
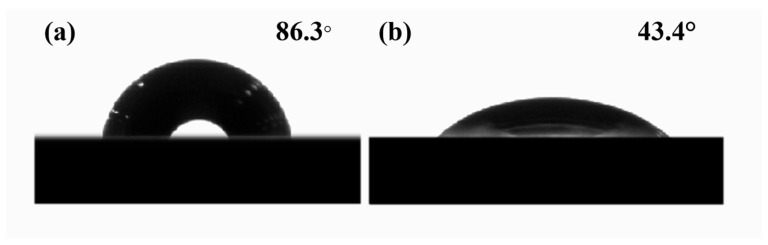
ACM results of the PC surface: (**a**) untreated; (**b**) after ablation, P_Ablation_ = 8 W.

To investigate the characteristics of chemical changes induced by laser ablation, this study employed XPS, Fourier transform infrared spectroscopy (FTIR), and scanning electron microscopy with energy-dispersive spectroscopy (SEM-EDS) to analyze the chemical composition of the PC surface before and after ablation. Compared to the pristine state, the characteristic peaks corresponding to the ester group (O-C=O) and carbonyl group (C=O) on the ablated PC surface, located at 289.0 eV and 288.0 eV, respectively, exhibited significant enhancement. In contrast, the characteristic peak associated with carbonate ester groups ((O)_2_-C=O) at 290.3 eV showed a marked decrease (as shown in Figure 7). These results indicate that laser ablation effectively reduces the content of carbonate ester functional groups on the PC surface while increasing the levels of ester and carbonyl functional groups. Additionally, analysis of the attenuated total reflectance-Fourier transform infrared spectrum (ATR-FTIR) spectrum (as shown in Figure 8) revealed intensified absorption bands corresponding to the stretching vibrations of C=O at 1728 cm^−1^ and C-O in the range of 1010–1289 cm^−1^ after ablation, further corroborating the increase in oxygen-containing functional groups. Notably, a new and more prominent absorption peak at 3500 cm^−1^, corresponding to hydroxyl groups (-OH), was observed on the ablated PC surface, in contrast to the pristine PC. This result suggests that laser ablation facilitates the formation of a substantial quantity of -OH groups, aligning with observations reported by A. Ramazani S.A et al. [30]. Furthermore, results from SEM-EDS demonstrated an increase in oxygen content on the ablated PC surface (as shown in Figure 9), indirectly indicating a higher concentration of oxygen-containing functional groups. The combination of these findings clearly demonstrates that femtosecond laser ablation induces oxidation reactions on the PC surface, leading to the deposition of numerous oxygen-containing functional groups. These groups are typically polar and hydrophilic, exhibiting greater polarity compared to carbonate ester groups (O)_2_-C=O. Subsequent WCA measurements revealed that the WCA of the ablated PC surface decreased from 86.3° to 43.4° (as shown in Figure 10). The improved wettability further supports the increase in surface polarity following ablation.

**Figure 11:**
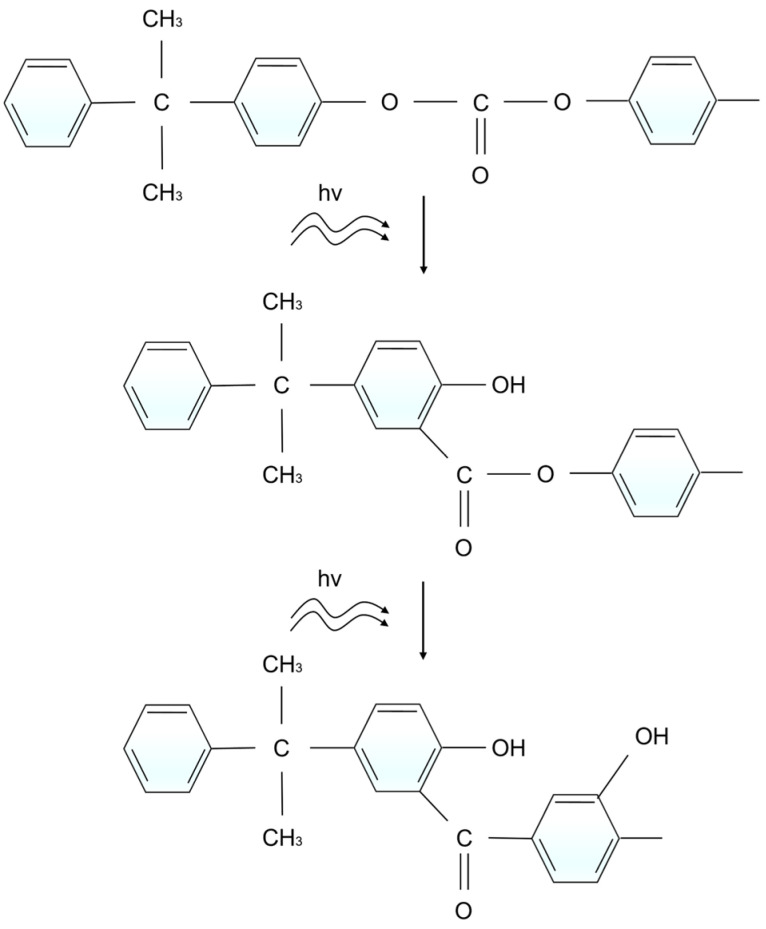
Photochemical rearrangement reaction scheme of PC.

In laser ablation, the photo-oxidative degradation reactions of polymers can be broadly divided into three stages: (a) molecular chains acquire sufficient energy to break, generating free radicals; (b) these free radicals rapidly react with oxygen atoms to form peroxyl radicals; (c) the resulting free radicals participate in crosslinking reactions, leading to the formation of new stable substances. Throughout this process, the involvement of oxygen results in an increased concentration of oxygen-containing functional groups on the polymer surface after ablation (as shown in Figure 8 and Figure 9). Previous studies have shown that photochemical reactions induced by ultrafast laser modification of PC exhibit a characteristic Light-Fries rearrangement effect, as illustrated in Figure 11. Under high-energy laser irradiation, (O)_2_-C=O can nonlinearly absorb two or more low-energy photons—primarily two—causing the cleavage of weak C-O bonds and the generation of free radicals. These free radicals subsequently undergo crosslinking reactions, converting (O)_2_-C=O into O-C=O or C=O, and producing phenyl salicylate and dihydroxybenzophenone as corresponding products [29,31,32]. Notably, a new strong absorption peak appears in the absorption spectrum of the modified products (as shown in Figure 8), indicating the formation of a substantial number of -OH groups after PC modification. The presence of numerous highly polar -OH groups further enhances the surface hydrophilicity.

### 3.2. Near-Infrared Welding Experimental Results and Analysis

**Figure 12:**
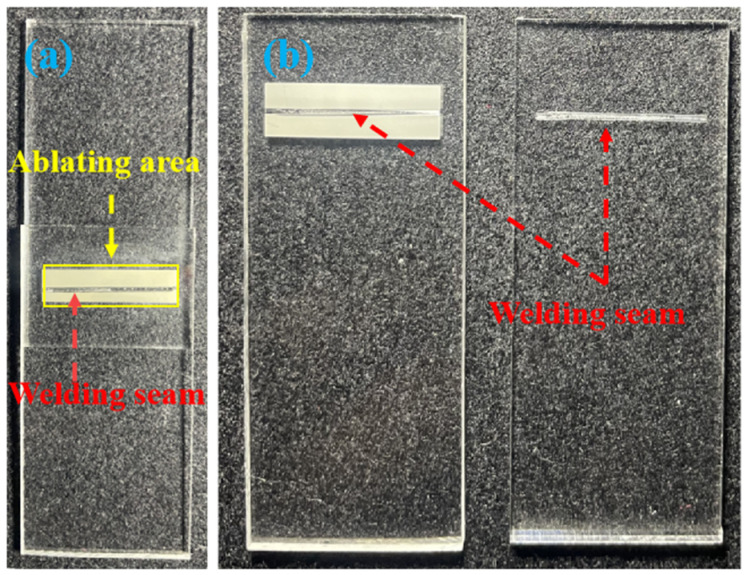
Pictures of the specimens tested in the shear test: (**a**) before the test; (**b**) after the test.

**Figure 13:**
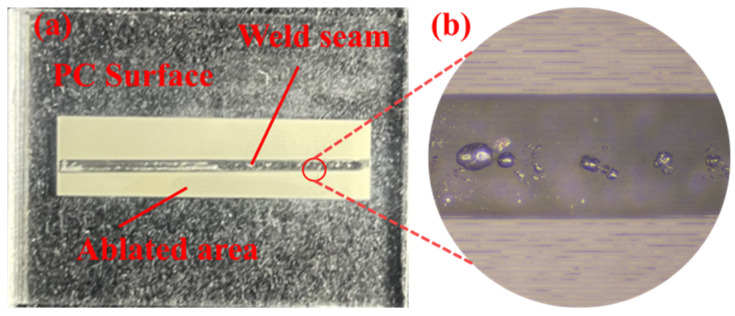
(**a**) Photograph of the welded sample and (**b**) close-up area of the weld seam (welding speed is 6 mm/s, P_Welding_ = 8.5 W, P_Ablation_ = 8 W).

**Figure 14:**
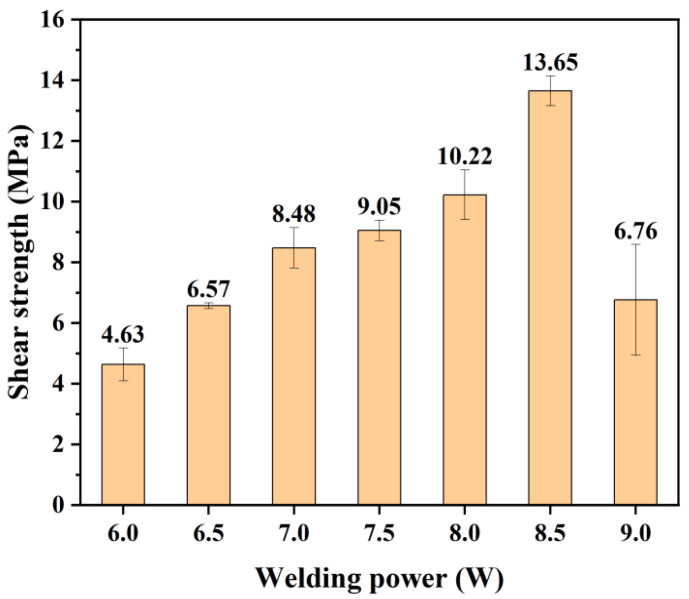
Variation in connection strength with increasing welding power (welding speed is 6 mm/s, P_Ablation_ = 8 W).

Under conditions without an absorbent, the physical appearance of PC and PS samples welded directly after ablation is shown in Figure 12a and Figure 13a (the local magnification of the weld seam is shown in Figure 13b), while the physical samples of the welded specimens after the tensile test are shown in Figure 12b. The modified region of the material was precisely controlled within predetermined limits, while areas outside this region remained intact and free of contamination. Notably, the modified section within the weld seam exhibits noticeable transparency to the naked eye. This transparency arose from the melting and recrystallization processes occurring in that region, where laser ablation-induced microstructures within the weld seam were eliminated, resulting in a translucent state. The presence of these microstructures is identified as a key factor contributing to enhanced transmittance. The bonding strength of weld joints at different welding powers is illustrated in Figure 14. A successful connection between PC and PS occurred when P_Welding_ = 6.0 W. Within the range of P_Welding_ < 9.0 W, increasing laser energy led to more pronounced diffusion and entanglement movements between the polymers, resulting in enhanced bonding strength for heterogeneous joints, with a maximum value of 13.65 MPa. However, when P_Welding_ reached 9 W, bubbles generated by thermal decomposition within the weld seam compromised the fatigue resistance of the joint, leading to a reduction in bonding strength for heterogeneous connections [33]. These results indicate that laser ablation not only facilitates the formation of microstructures that can substitute for photo absorbers but also enables effective direct connections between otherwise unweldable PC and PS materials. The welding performance between dissimilar polymers is closely related to their solubility parameters *δ*, which include dispersive forces δ_d_, polarity δ_p_, and hydrogen bonding δ_h_. The solubility parameters of PC, PS, and their respective components are provided in Table 2.

**Figure 15:**
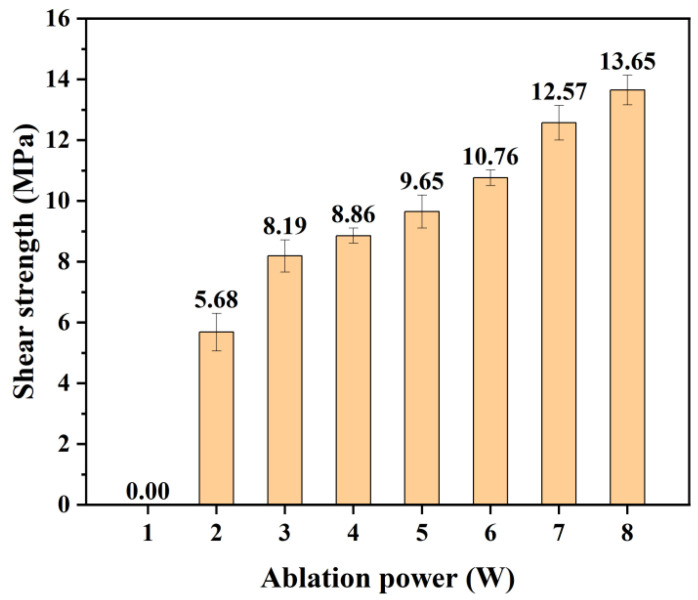
The variation in bonding strength with increasing ablation power (welding speed is 6 mm/s, P_Welding_ = 8.5 W).

The numerical values of the solubility parameters of liquids are calculated by the Hansen solubility parameters formula (Equation (3)). After the polymer/polymer mixture, the enthalpy of the solution per unit volume will change, and the change value (∆h_M_) can be calculated by Equation (4). According to the thermodynamics of the blending system, the smaller the change in free energy (∆F_m_, calculated by Equation (5)) before and after the mixture of different solutions (including molten polymers), the better the solution mixing effect. From Equations (4) and (5), it can be known that when the values of each component of the solubility parameters of two liquids (δ_d_, δ_p_, δ_h_) are close to each other, ∆hM will decrease, and the mutual solubility of the two solutions will become better. This rule is called “like seeks like”, which physically reflects that substances with similar types and magnitudes of intermolecular forces in each component have better mutual solubility. Therefore, according to the Hansen solubility theory, the closer the values of each component of the solubility parameters of the mixed solution, the better their compatibility and connection performance [16]. As illustrated in Table 2, significant differences exist in the polarity and hydrogen bonding components between PC and PS, which indicates weaker van der Waals forces between these materials. This difference is a key factor contributing to their inability to be directly welded. In this study, femtosecond laser ablation technology was employed to enhance the polarity of PC, thereby reducing the disparity in polarity components between PC and PS. This improvement enhanced the diffusion and entanglement capabilities of dissimilar polymers, ultimately resulting in better miscibility and bonding performance. Under the optimal welding parameters (P_Welding_ = 8.5 W), the effect of varying ablation power (P_Ablation_) on the bonding performance of PC and PS was further investigated. As illustrated in Figure 15, within the range of 2 W ≤ P_Ablation_ ≤ 8 W, a strong positive correlation exists between the bonding strength of the heterogeneous joint and the ablation power.

(3) $$\delta^{2}=\delta_{d}^{2}+\delta_{p}^{2}+\delta_{h}^{2}$$

(4) $$\Delta h_{M}=\varphi_{1}\varphi_{2}\left[{\left(\delta_{d1}-\delta_{d2}\right)}^{2}+{\left(\delta_{p1}-\delta_{p2}\right)}^{2}+{\left(\delta_{h1}-\delta_{h2}\right)}^{2}\right]$$

(5) $$\Delta F_{M}=\Delta H_{M}-T\Delta S_{M}$$

The influence of ablation power on welding performance primarily involves both physical and chemical mechanisms. On the physical side, an increase in ablation power raises the surface roughness, which directly enhances the laser absorption capacity of the material. This suggests that, under constant welding power, a more textured welding surface can absorb greater amounts of laser energy, promoting more effective molecular interdiffusion and entanglement. On the chemical side, the specific surface area in the modified region (as shown in Figure 4a3–h3) and the density of oxygen-containing functional groups (as shown in Figure 9) both increase with higher ablation power. This leads to a corresponding increase in the content of induced functional groups, which further improves miscibility and bonding performance between dissimilar materials. In summary, experimental results demonstrate that laser ablation can significantly enhance the welding performance of PC and PS. By adjusting the ablation power, it is possible to effectively control the bonding strength of heterogeneous joints. In recent years, with the rapid development of the lightweight trend in construction and transportation vehicles such as automobiles or aircraft, the combined use of various polymers and their composites with excellent properties and high specific strength will be widely applied and promoted. This process provides a new potential solution for the efficient connection and assembly of these materials. Therefore, the research on ablation technology in the field of welding of dissimilar polymers will be given due attention.

## 4. Conclusions

To address the challenges of low light absorption and poor weldability, this study introduced a novel strategy to enhance the welding performance of dissimilar transparent polymers using femtosecond laser surface modification technology. Experiments were conducted on femtosecond laser ablation of transparent polymers at a wavelength of 520 nm, as well as on the near-infrared LTW of dissimilar transparent polymers. The investigations included characterization and mechanical property testing, with a primary focus on analyzing the effects of ablation power and laser power on the welding performance of PC and PS. A comprehensive analysis was carried out to elucidate the mechanisms by which laser ablation-induced processes improved the welding capability of dissimilar transparent polymers. The key findings can be summarized as follows:

(1) The microstructures generated by laser ablation significantly influence surface roughness and light absorption properties, demonstrating a strong positive correlation with ablation power. As P_welding_ increases from 1 W to 8 W, the surface roughness and light absorption rate increase from 0.61 μm to 5.49 μm and from 12.73% to 32.35%, respectively.
(2) These microstructures effectively replaced absorbents in the LTW process, enabling direct welding of transparent polymers using near-infrared lasers without the need for additional absorbents.
(3) Laser ablation promoted the formation of oxygen-containing functional groups (C-O, C-O, O-C-O, -OH), which enhance the miscibility and wettability of dissimilar polymers, thereby improving their interfacial bonding performance despite their initial non-weldability.
(4) Both welding power and ablation power were critical determinants of the welding strength of heterogeneous joints, with a maximum welding strength of 13.65 MPa achieved in the experiments.

## Figures and Tables

**Table 1:** The physical properties of PC and PS.

Materials	Density (g/cm³)	Shear Strength (MPa)	Transmittance (%)	
			520 nm	808 nm
PC	1.194	54.1	88.3	90.05
PS	1.06	56.8	89.9	90.28

**Table 2:** Data for PC and PS are derived from the literature [34,35].

Material	δ_d_ (MPa^1/2^)	δ_p_ (MPa^1/2^)	δ_h_ (MPa^1/2^)	Δ (MPa^1/2^)
PS	19.1	13.3	1.0	23.3
PC	18.1	5.9	6.9	20.2

## Data Availability

The original contributions presented in this study are included in the article. Further inquiries can be directed to the corresponding author.
